# FRET Microscopy in Yeast

**DOI:** 10.3390/bios9040122

**Published:** 2019-10-11

**Authors:** Michal Skruzny, Emma Pohl, Marc Abella

**Affiliations:** 1 Department of Systems and Synthetic Microbiology, Max Planck Institute for Terrestrial Microbiology, 35043 Marburg, Germany; 2 LOEWE Center for Synthetic Microbiology (SYNMIKRO), 35043 Marburg, Germany

**Keywords:** budding yeast, fission yeast, acceptor photobleaching, sensitized emission, ratiometric FRET, FLIM, GFP

## Abstract

Förster resonance energy transfer (FRET) microscopy is a powerful fluorescence microscopy method to study the nanoscale organization of multiprotein assemblies in vivo. Moreover, many biochemical and biophysical processes can be followed by employing sophisticated FRET biosensors directly in living cells. Here, we summarize existing FRET experiments and biosensors applied in yeasts *Saccharomyces cerevisiae* and *Schizosaccharomyces pombe*, two important models of fundamental biomedical research and efficient platforms for analyses of bioactive molecules. We aim to provide a practical guide on suitable FRET techniques, fluorescent proteins, and experimental setups available for successful FRET experiments in yeasts.

## 1. Introduction

**Figure 1:**
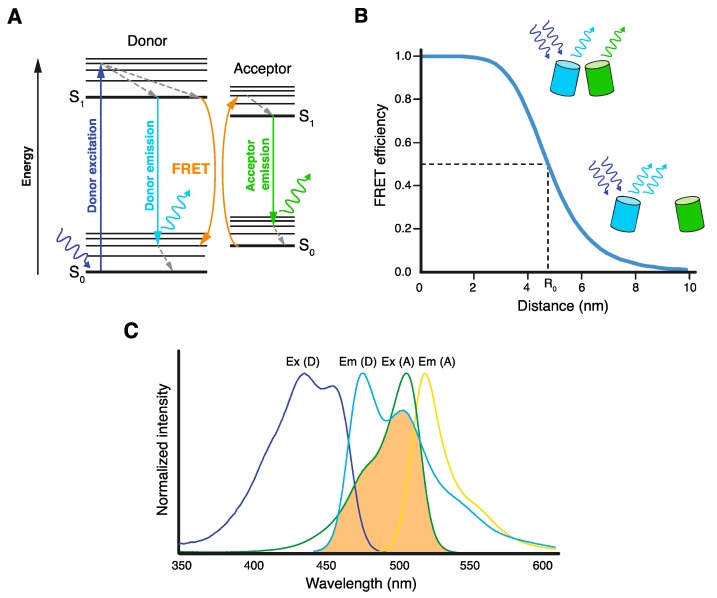
Basic principles of Förster resonance energy transfer (FRET). (**A**) Jablonski diagram illustrating excitation and emission of the donor fluorophore and FRET between the donor and acceptor fluorophores, resulting in acceptor emission. (**B**) Dependence of FRET efficiency on the distance *R* between donor and acceptor molecules. Förster radius *R*_0_ is the distance at which half of the energy of the excited donor is transferred by FRET. (**C**) Excitation/absorption (Ex) and emission (Em) spectra of mTurquoise2 donor (D) and mNeonGreen acceptor (A) FRET pair. Spectral overlap between mTurquoise2 emission and mNeonGreen excitation spectra, which is essential for FRET, is highlighted in orange.

Förster resonance energy transfer (FRET) is an electromagnetic phenomenon, by which the energy of a light-excited fluorophore molecule (FRET donor) is transferred in a non-radiative way (by dipole–dipole coupling) to another molecule (FRET acceptor) located in very close proximity [1,2,3]. FRET leads to decreased fluorescence emission of the FRET donor, manifested either by its lower intensity or shorter lifetime. When the FRET acceptor is also a fluorophore, FRET leads to its excitation and subsequent fluorescence emission (Figure 1A). In addition, FRET changes the polarization of the fluorescence emitted by both donor and acceptor.

As described in the following three fundamental equations, FRET efficiency (*E*_FRET_) depends on the orientation of donor and acceptor dipole moments (*κ*^2^), the quantum yield of the donor (Φ_0_), the extinction coefficient of the acceptor (*ε_A_*), the overlap integral between the normalized donor emission and acceptor excitation spectra (*J*), and, very importantly, the sixth root of the distance between donor and acceptor molecules (*R*) [1,2,3,4]:

(1) $$E_{FRET}=\frac{R_{0}^{6}}{R^{6}+R_{0}^{6}}$$

(2) $$R_{0}^{6}=0.021J\kappa^{2}\Phi_{0\;}n^{-4}$$

(3) $$J=\int^{\;}{\bar{I}}_{D}\varepsilon_{A}\lambda^{4}d\lambda$$

with ${\bar{I}}_{D}$ being the normalized donor emission intensity, *n* the refractive index of the medium, and *λ* the wavelength. *R*_0_ is the Förster radius (in nm), a distance specific to every donor–acceptor pair, at which an excited donor releases half of its energy by FRET (*E*_FRET_ = 0.5).

Because of its strict distance dependency, FRET usually occurs only between molecules separated by less than 10 nm (Figure 1B). This endows FRET with a unique nanometer sensitivity, which is very advantageous for its application to study proximities and interactions of various biomacromolecules, either in vivo or in vitro [4,5,6]. Especially in living cells, several FRET microscopy techniques were implemented to study (supra)molecular structures, events, and reactions with unprecedented spatial resolution, and with only minimal interference with the studied system [7,8]. The prerequisite for in vivo FRET experiments is tagging the molecules of interest with suitable FRET donor and acceptor, either chemically or genetically [9,10]. One of the most common techniques is the attachment of FRET-proficient fluorescent proteins (FPs) to proteins of interest via the co-expression of their coding sequences in cells. With the constant improvement of suitable FPs, tagging, illumination, and detection techniques, FRET microscopy has become increasingly popular among researchers studying various aspects of life at the molecular level.

Though there are many biological questions that can be powerfully approached by FRET microscopy in vivo, for simplicity we split here these efforts into two broad areas: (i) studies on spatiotemporal organization of cellular multiprotein assemblies using FRET as a molecular ruler, and (ii) analyses of biochemical and biophysical reactions in cells using FRET biosensors. We will also narrow our focus to FRET studies performed with two important unicellular model organisms: budding yeast *Saccharomyces cerevisiae* and fission yeast *Schizosaccharomyces pombe*. Both yeasts have continuously served as “living test tubes” for studies of fundamental cellular processes, and as “screening platforms” for analysis of various bioactive compounds. Their pivotal role in current biomedical research is justified by many molecular processes conserved between them and humans, their straightforward cultivation and genetic manipulation, and their exceptionally well-characterized molecular biology and biochemistry. As summarized below, FRET microscopy has been a very beneficial method leading to many important discoveries made with yeasts.

To strengthen the application of FRET microscopy in yeast, we first provide an overview of FRET experiments performed to map yeast multiprotein assemblies in vivo. Second, we list FRET biosensors applied to study biochemical or biophysical events in yeast. Third, we describe suitable FRET techniques and fluorescent proteins for FRET microscopy in yeast, discussing their advantages and potential shortcomings. Finally, we provide several practical tips for successful FRET imaging of yeasts cells. We assume that also readers not directly working with yeast will find this review helpful, as many topics covered (choice of suitable FRET technique, fluorophores, etc.) are broadly applicable to FRET experiments with other uni- and multicellular organisms.

## 2. Mapping the Organization of Yeast Protein Complexes by FRET

**Figure 2:**
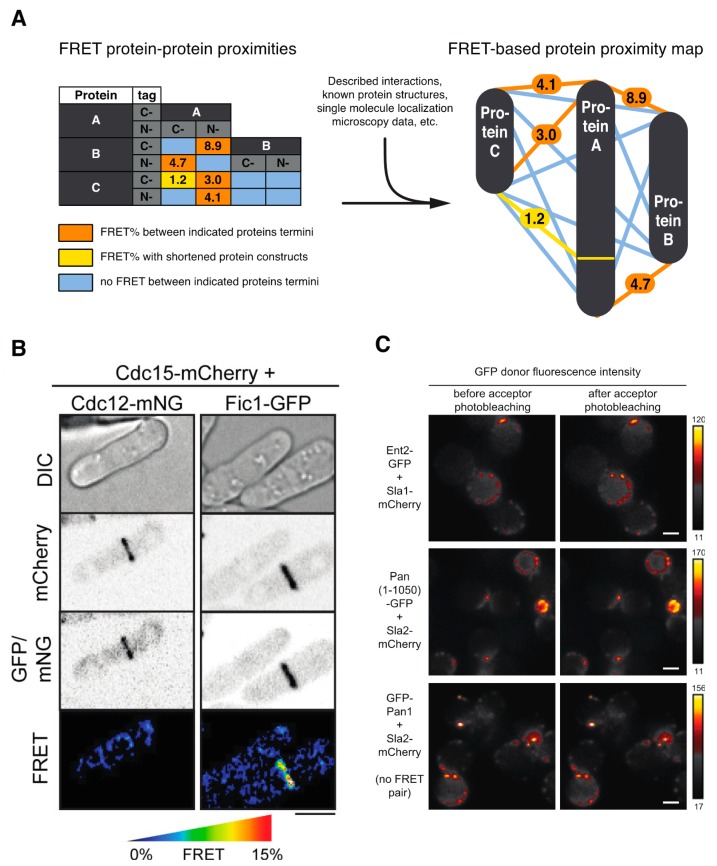
Mapping the organization of yeast protein complexes with FRET. (**A**) General scheme of FRET-based protein proximity mapping between proteins A, B, and C, tagged with fluorescent proteins (FPs) either N- or C-terminally. (**B**) No FRET and FRET between indicated FP-fusion proteins of *Schizosaccharomyces pombe* cell division contractile ring. See [21] for further details. (**C**) FRET between indicated FP-fusion proteins of *Saccharomyces cerevisiae* endocytic coat complex is shown as an increase of green fluorescent protein (GFP) donor fluorescence intensity after mCherry acceptor photobleaching. See [22] for further details. (**A**,**C**) Images adopted from [22]; (**B**) image taken from Figure 5 of [21]. Scale bars: 4 μm (**B**), 2 μm (**C**).

The characterization of the nanoscale architecture of cellular supramolecular assemblies is critical for the understanding of their function and mechanism of action. Because of its unique spatial sensitivity, FRET has been repeatedly used to elucidate the organization of many crucial multiprotein complexes in yeast. In a general scheme of these experiments (Figure 2A), yeast strains containing pairs of complex subunits tagged with donor and acceptor FPs are first constructed and tested for functionality. Cells are then analyzed for the presence of FRET between the protein fusions, which indicates their molecular proximity. Further views of a complex arrangement or its potential regulation can be then acquired by using truncated or mutated proteins, or by working with cells deleted of potential cofactors, bridging proteins, modifying enzymes, etc.

The molecular architecture of several large multiprotein assemblies, which consist of dozens of subunits (usually presented in multiple copies), has been systematically analyzed by FRET in yeast: the organization of the nuclear pore complex and its associated transport factors [11,12], kinetochore [13,14,15], spindle pole body (microtubule organizing center in yeast) [16,17,18,19,20], cell division contractile ring [21] (Figure 2B), and endocytic coat [22,23] (Figure 2C). Also, interactions of several smaller complexes of DNA/chromatin regulatory proteins were determined by FRET: the architecture of yeast cohesin [24], interaction between Gal4 transcription factor and SAGA complex [25], crosstalk between PCNA protein Pol30 and SAS-I complex [26], or binding of ATR kinase complex Dcp2-Mec1 with PP4 phosphatase Psy2-Php3 [27]. The assembly of membrane-associated complexes, including the oligomerization of protein Ste2 [28,29,30], iron permease Fet3-Ftr1 [31], copper transporter Ctr1 [32], and Ato1-Ato2 proteins [33]; Tom70 fragment [34]; and vacuolar V-ATPase [35], was followed by FRET on the yeast plasma, mitochondrial, and vacuolar membranes, respectively. Importantly, several complexes of principal signaling pathways were assessed by FRET microscopy in yeast: interaction of cyclin-dependent kinase inhibitor Sic1 with various cyclins [36] or binding of Ste5 scaffold with Fus3 MAPK kinase during activation of the yeast mating pathway [37]. Finally, FRET has also been used to map interactions of heterologous proteins expressed in yeast. Very interesting examples of this approach are studies on disease-related aggregation of Prp prion alone or with amyloid β peptide [38,39], hungtingtin protein [40], or yeast protein Toh1 with yeast prions Rnq1 and Sup35 [41]. The protein complexes mapped by FRET in yeast are summarized in Table 1, also highlighting the used FRET fluorophores and applied techniques. Several of these studies resulted in a specific FRET protocol for a given complex or compartment (see Table 1).

These successful efforts illustrate the power of FRET-based mapping of protein assemblies in yeast. The main advantage is the simplicity by which yeast strains expressing FP-tagged pairs of complex subunits can be generated. The protein fusions can be made either N- or C-terminally, and very importantly, are usually expressed from endogenous loci, assuring near-native protein levels. Though this could be eventually reached in other cell types too (e.g., by CRISPR-Cas9 DNA editing techniques), the following tests for functionality of constructed protein fusions are very straightforward in yeast haploid cells.

FRET-based protein proximity mapping in yeast can also encounter some difficulties. Aside from constraints not directly related to FRET (e.g., the given protein complex is evolutionarily too dissimilar to be studied in yeast), the densely packed yeast cytoplasm and highly mobile intracellular membrane compartments could be disadvantageous for FRET mapping of certain protein complexes. Fluorescently tagged subunits of low abundance, or inversely, highly ubiquitous protein complexes, might give an insufficient signal-to-noise ratio of fluorescence signal, making their FRET analyses difficult. In addition, protein complexes of mobile membrane organelles might be too dynamic for reliable FRET measurements. Nevertheless, further developments of fluorescent microscopy tools might soon provide better spatiotemporal resolution for FRET analyses of these low-abundant or dynamic yeast protein complexes.

## 3. Analyzing Biochemistry and Biophysics of Yeast by FRET Biosensors

**Figure 3:**
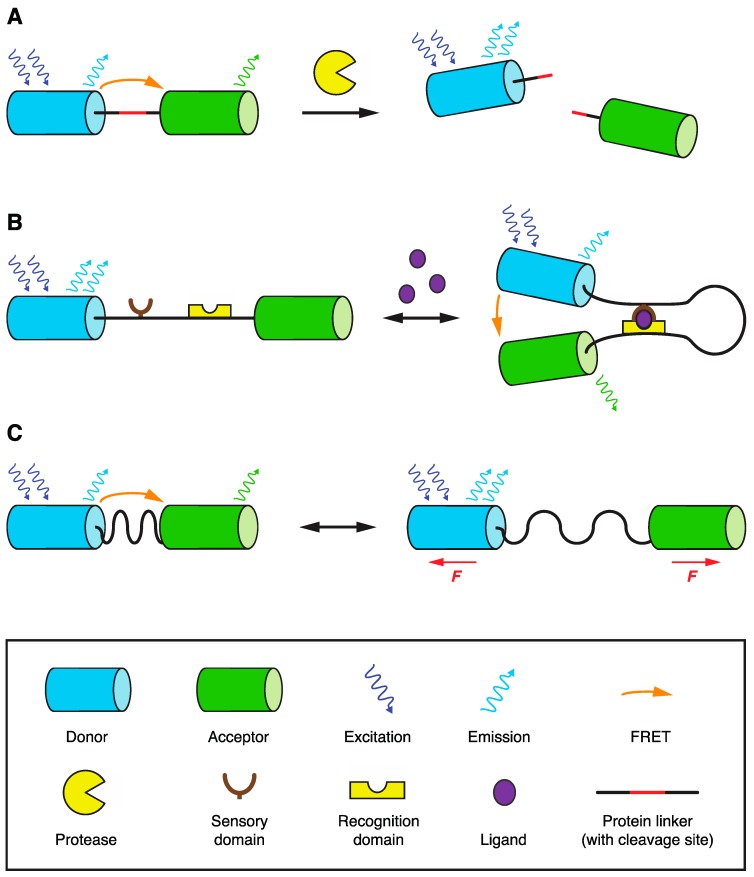
Common types of FP-based FRET biosensors. In these sensors, change in distance (or orientation) of appended FPs is followed by change in FRET between them. This can be used to monitor: (**A**) protease activity causing the cleavage of a particular linker between FPs; (**B**) presence of ligand or posttranslational modification on the sensory part of the sensor, which is recognized by other parts of the sensor, causing its conformational change; (**C**) molecular force/tension applied over the sensor inserted in force-bearing protein. See legend at the bottom part of the figure for further details. Adopted from [47].

Genetically-encoded FRET biosensors became popular tools for monitoring various molecular events in vivo soon after the first application of fluorescent proteins [45]. Though diverse in their molecular design, the unifying theme of FRET biosensors is the presence of a FRET donor and acceptor pair reporting about a specific molecular event by changes in their proximity or orientation, hence in FRET. These changes can be invoked by several means: by sensor cleavage, by conformational change of the sensor after ligand/cofactor binding, by chemical modification, by physical stretching of the sensor, etc. [46,47] (Figure 3). The donor and acceptor parts of a FRET biosensor can be either attached to two separate molecules or be part of a single macromolecule. The later setup is advantageous as it greatly simplifies the FRET readout because of the equimolar concentration and identical spatial behavior of the donor and acceptor.

Though development of FRET biosensors is often a complex endeavor [48], they offer many advantages in comparison to other biochemical and biophysical analytical tools. Foremost, they can be used in living cells with only minimal perturbations of the studied system [49]. Next, because of their fluorescence readout, they provide high sensitivity to analyze a given reaction, either at the population, single cell, or even subcellular level. Finally, as FRET signal of a sensor can usually be followed over time, various modulations of the studied reaction can be directly performed and analyzed in vivo (e.g., changing the concentration of reactants, temperature, activity of involved enzymes).

Table 2 summarizes FRET biosensors applied in yeast so far. Many sensors have been used to follow metabolites and ions fluxes in yeast. The design, calibration, and application of this type of FRET sensors have already been carefully discussed [49], so we provide here an update of more recently implemented sensors (see Table 2). These sensors not only assess metabolite/ion concentrations in yeast, but also help to delineate functions and regulatory networks of involved enzymes (e.g., in respective mutant strains) [50,51,52]. Remarkably, some metabolite/ion FRET biosensors were directly developed from specific yeast proteins or systematically optimized in yeast. To the first group belong zinc ion, copper ion, and redox FRET biosensors based on parts of transcription factors Zap1, Ace1 (or *Candida albicans* homolog Amt1), and Yap1, respectively [53,54,55]. The second group is represented by FRET biosensors for nitrate and oligopeptide transport in plants, and for the plant hormone abscisic acid [56,57].

As mentioned in the previous section, FRET can be efficiently used to study medically related prion aggregation in yeast. In a pilot study, an elegant FRET biosensor (AmFRET) was designed to analyze nucleation kinetics of several yeast prion-like proteins [58]. It consists of a single photoconvertible FP, mEos3.1, fused to the studied protein. When mEos3.1 was partially photoconverted from its green to red form by blue light, the two mEos3.1 forms became the FRET donor and acceptor, showing FRET during protein aggregation. In addition, yeast was also used as a screening platform for the evolution of proteases specifically targeting amyloid β peptide, here fused to CyPet-YPet FPs for FRET readout of its cleavage [59] (see scheme in Figure 3A).

Aside from tracing individual molecules, FRET biosensors can also monitor crucial cellular processes. Transcriptional activity of different RNA polymerase II promoters was followed by the expression of RNA aptamers (IMAGEtags), which mediate FRET of its specific Cy3/5-labeled ligands [60]. Activities of principal signaling pathways, mating MAPK kinase and starvation cAMP/PKA pathways, were analyzed by FRET sensors for MAPK activity (yEKAREV) [61], cAMP presence (Epac2-camps), and PKA activity (AKAR3) [62]. The kinase activity sensors contain a kinase-specific phosphorylation site connected by a flexible linker to a particular phosphopeptide-binding domain. The intramolecular binding of these two moieties is monitored by FRET changes between FPs also carried by the sensor (see scheme in Figure 3B).

Finally, the biophysics of several cellular processes has been studied by sophisticated FRET-based tension/force biosensors in yeast (see scheme in Figure 3C). Force applied on the kinetochores during chromosome segregation was recently analyzed by a FRET tension sensor incorporated into the kinetochore protein Ndc80 [63]. Experiments are also ongoing to analyze forces required for plasma membrane invagination during endocytosis. Here, calibrated FRET-based tension sensors (originally developed for mechanotransduction studies of focal adhesions [64]) were introduced into force-transmitting protein Sla2 to monitor forces generated by the actin cytoskeleton during endocytic vesicle formation in yeast [65,66].

The advantages and shortcomings of FRET biosensors in yeast are similar to pros and cons of FRET-based protein–protein mapping. Several expression systems can be applied to regulate the expression of genetically-encoded FRET sensors in yeast. Attention should again be paid to control for the specificity of the FRET signal, especially in the case of very low or high expression of sensors. However, when monitoring of FRET biosensors is established, the ease of genetic and experimental manipulations of yeast opens many possibilities for a potential interference and subsequent analysis of studied events, either in single cells or cell population.

## 4. FRET Microscopy Techniques for Yeast Models

**Figure 4:**
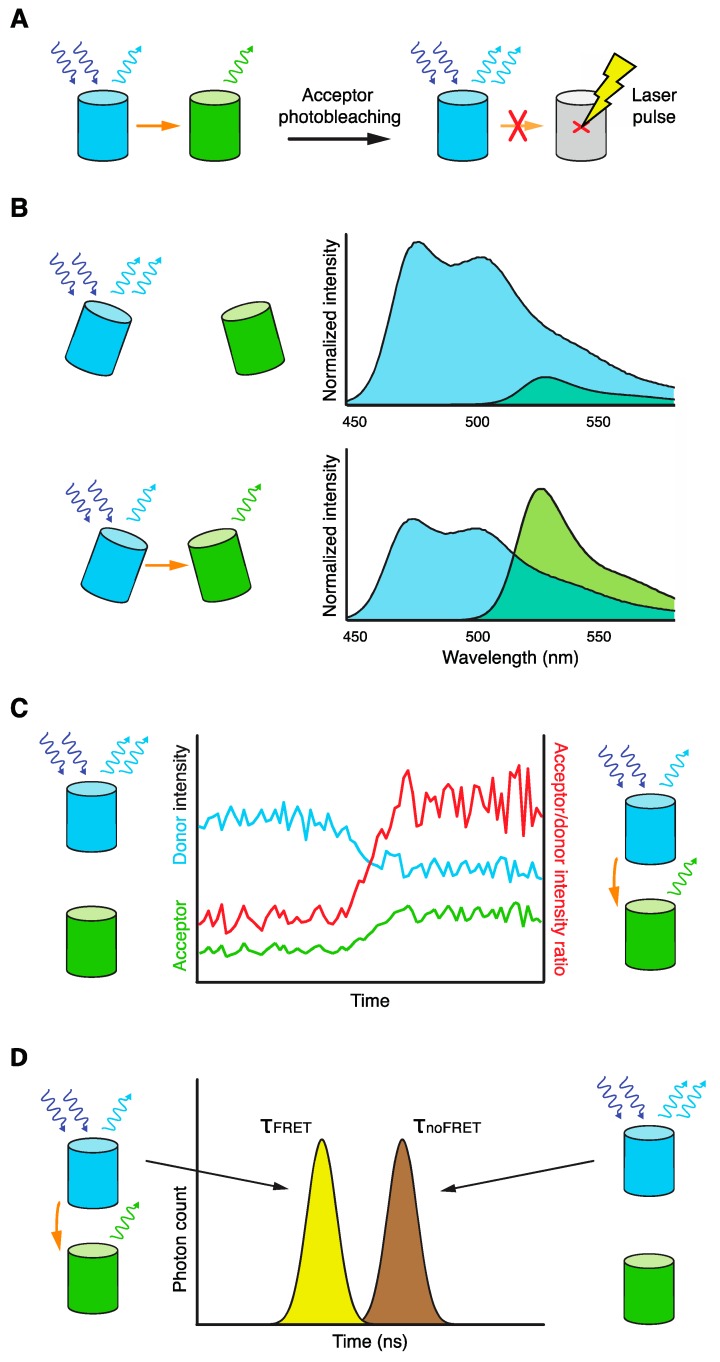
Basic principles of FRET microscopy techniques. (**A**) Acceptor photobleaching. Increase in donor fluorescence after the photo-inactivation of acceptor FP by a laser signalizes FRET. (**B**) Sensitized emission. FRET causes enhanced emission of acceptor fluorescence and decreased emission of donor fluorescence. These changes can be measured either over the whole spectrum (spectral FRET) or in specific wavelength windows (tri-cube FRET). (**C**) Ratiometric FRET. The simplest readout of sensitized emission, when the ratio of acceptor/donor fluorescence is followed over time. (**D**) FRET-FLIM. FRET decreases the fluorescence lifetime of the donor. Pools of FRET-involved and FRET-absent donor molecules are discernible by their different fluorescence lifetimes.

The principles of FRET allow its measurement by several different microscopy or spectroscopy techniques [3,4,8,77,78,79,80,81,82,83,84]. They can be divided into methods following changes in donor or acceptor fluorescence intensity, donor or acceptor fluorescence polarization/anisotropy, and the lifetime of the donor fluorescence (Figure 4).

The presence of FRET lessens the fluorescence emitted by excited donor molecules. This can be observed either as quenching of donor fluorescence in the presence of an acceptor, or its dequenching in the acceptor’s absence. The later phenomenon can be easily achieved without actual removal of the acceptor by its specific photoinactivation with a laser, giving the method its name: donor dequenching after acceptor photobleaching, or more simply, acceptor photobleaching (Figure 4A). Acceptor photobleaching represents a simple and reliable FRET technique that can be easily performed with any fluorescence microscope equipped with a laser suitable to photobleach the chosen acceptor FP. The experimental setup and analysis are straightforward: Several acceptor and donor acquisitions are taken before and after acceptor photobleaching. The completeness of acceptor photobleaching is controlled and donor fluorescence is corrected for structural photobleaching occurring during the acquisition. The FRET efficiency is then calculated as percentage increase of donor fluorescence after acceptor photobleaching in comparison to its prebleach value. Because of the size of yeast cells, several cells can usually be efficiently photobleached in a short time without any phototoxic effects. A critical drawback of this method is its endpoint character caused by irreversible photodestruction of the acceptor fluorophore. Nevertheless, FRET changes in time (e.g., expected change of protein complex composition or conformation) can be followed indirectly, for example by using another protein as a “time stamp” of the process (see [19,22] for examples). To capture transient molecular proximities, cells can first be fixed with formaldehyde, which was recently shown to preserve temporal protein complexes, as well as FRET between FPs [22,85].

By means of FRET, acceptor molecules can be excited, releasing the acquired energy as fluorescence (Figure 4B). This is called donor-sensitized acceptor emission (or simply sensitized emission), which can be observed by many standard wide-field and confocal microscopes equipped with filter sets for chosen fluorophores. Sensitized emission can be registered in setups dubbed “three-cube FRET” and “spectral FRET”. For the common “three-cube FRET” technique, three different acquisitions of the sample are taken using filters for: donor excitation and emission, acceptor excitation and emission, and donor excitation and acceptor emission (so-called “FRET channel”). Next, acquisitions of donor- and acceptor-only cells are performed to correct for the bleed-through of donor emission into the acceptor emission channel and acceptor cross-excitation after donor excitation, respectively. In the end, three different strains should be imaged next to each other to avoid day-to-day variations in protein expression, microscopy setup, etc. Though the acquisition is then slightly more complex and error-prone in comparison to acceptor photobleaching, the calculation of the apparent FRET efficiency is again relatively simple, as it is already implemented in many microscopy and image analysis software tools. Sensitized emission can be similarly determined by “spectral FRET” recording full emission spectra after donor and acceptor excitation [77]. The key advantage of both techniques is the option of (semi-)continuous FRET measurements over time. This is even more apparent for the simplified version of sensitized emission recording, “ratiometric FRET”. Here, only ratios of acceptor and donor emission (or vice versa), invoked by donor excitation, are followed during a biological process or after its experimental intervention (substrate or inhibitor addition, etc.). The ratiometric readout also has a higher sensitivity to follow FRET changes in comparison to monitoring donor or acceptor signals separately (Figure 4C). Donor and acceptor emissions can be recorded either sequentially by changing microscope filter cubes, or simultaneously using an image splitting device containing both emission filters and an appropriate dichroic mirror. The simplicity and speed of recording makes ratiometric FRET the method of choice for analyses of relative FRET changes in real time (e.g., of calibrated FRET biosensors).

The presence of a FRET partner also changes the anisotropy (polarized character) of the fluorescence emitted by the donor/acceptor when the polarized light is used for its excitation. Techniques following fluorescence anisotropy are highly sensitive for FRET and offer fast readout (useful for high-throughput screens), but they are not quantitative. Importantly, they can be applied on a pool of molecules tagged with a single FP, where individual FP molecules work both as donor and acceptor because of an overlap between FP’s excitation and emission spectra. This special technique, called “homoFRET”, is often used to analyze clustering or homo-oligomerization of tagged molecules, as further detailed in [83].

Finally, in the presence of a FRET acceptor, donor molecules stay shorter in their excited state (as their energy relaxation by FRET is substantially faster than the relaxation by photon emission), which is connected to the shorter lifetime of their fluorescence. This can be followed by sophisticated techniques of fluorescence lifetime imaging microscopy (FLIM, or more precisely, FRET-FLIM), which compare changes in donor fluorescence lifetime in the presence or absence of an acceptor (Figure 4D). FRET-FLIM techniques follow either fluorescence decay of donor molecules simultaneously excited by a pulse laser (time domain measurements) or phase shifts of donor-emitted fluorescence (frequency domain measurements) [81,82]. Pools of donor molecules involved in—or absent of—FRET can be discerned by FRET-FLIM because of their different fluorescence lifetimes (Figure 4D). In addition, FRET-FLIM is also much less sensitive to concentration differences between donor and acceptor molecules in comparison to other FRET techniques. Altogether, FRET-FLIM offers deeper mechanistic insights into the analyzed FRET system at the expense of specialized microscopy instruments and complex analysis of acquired data. Theoretically, only two measurements are necessary: determination of the donor fluorescence lifetimes in strains containing and absent of an acceptor. Practically, several other controls are often needed to properly calibrate a sensitive FRET-FLIM system (cells with free donor FP, donor–acceptor fusion, etc.). In addition, to obtain statistically robust fluorescence decay data, thousands of emitted photons need to be registered. This usually requires a high number of donor molecules being recorded for a certain time, so low abundant proteins might not be suitable for FRET-FLIM. In conclusion, if not directly available in an adjacent expert laboratory or core facility, FRET-FLIM techniques are rather suited as an advanced step to study FRET systems already explored by less-elaborate FRET methods mentioned above.

The advantages and potential drawbacks of discussed FRET methods, as well as instrumental and analytical requirements, are summarized in Table 3.

## 5. Fluorescent Proteins and Imaging Tips for FRET Microscopy in Yeast

The choice of FPs used as the FRET donor and acceptor is very important for a successful FRET experiment. As already mentioned, FRET depends on the quantum yield of the donor and the extinction coefficient of the acceptor, two key characteristics constituting the in vitro brightness value of FPs. In addition, a substantial overlap between the donor emission and acceptor excitation spectra is critical for FRET. Several other FP characteristics are, however, important for its in vivo performance, especially for FRET measurements [47,86,87]. Foremost, it is essential to assess the practical in vivo brightness of FP in yeast, which is influenced by its folding and maturation time, pH and ion sensitivity, degradation rate, etc. Next, for FRET time-lapse experiments, the photostability of FPs is very important, as accumulation of photobleached FRET-inactive molecules decreases the FRET sensitivity and can influence the calculated FRET values. Finally, only monomeric FPs with simple decay kinetics and that are absent of photochromic effects should be used to avoid artifacts in FRET calculations [47,86,87].

Advantageously, yeast biologists have already tested and implemented many FPs for yeast in vivo imaging studies. These efforts culminated in the recent comprehensive study that compared important characteristics of many advanced versions of FPs in yeast [88]. Based on the current literature and our experimental expertise, we suggest below the most suitable FRET pairs and discuss their usefulness for various FRET approaches. Nevertheless, as development of new FPs continues, other combinations of FPs suitable for FRET in yeast will certainly be achievable after rigorous in vivo validation. To search for other FPs, we suggest the use of FPbase (www.fpbase.org), a curated database of available FPs, which also provides an intuitive, user-adoptable FRET calculator [89].

Because of their good spectral overlap and advantageous match of their brightness, cyan fluorescent proteins (CFPs) and yellow fluorescent proteins (YFPs) have often been used for FRET analyses, not only in yeast (see Table 2). This applies not only for traditional enhanced CFP and YFP (ECFP–EYFP) FRET donor–acceptor pair, but also for later implemented advanced CFP and YFP variants: mCerulean, CyPet, mTurquoise2, and mVenus, mCitrine, YPet, respectively. The limitations of CFP-YFP FRET pairs are: (i) relatively low brightness of CFPs, which together with high autofluorescence of yeast cytoplasm below 490 nm, decreases signal-to-noise ratio of their fluorescence, especially in the case of low-abundant proteins and sensors; (ii) some unwanted characteristics of YFPs, including their lower photostability, higher pH/ion sensitivity, and dimerization tendencies. Though not much brighter than ECFP, we suggest mTurquoise2 (mTq2) [90] as CFP-like donor because of its better signal-to-noise readout for several tested yeast protein structures (Milani et al.; in preparation). A very good substitute to YFPs is the green-yellow fluorescent protein mNeonGreen (mNG) [91], which in yeast is a very bright, fast-folding, and photostable monomeric FP with simple decay kinetics [88,92]. The blue shift of its excitation spectrum also increases its spectral overlap with CFPs. According to comprehensive tests in other systems [93] and in our practice [22,66] in yeast, mTq2-mNG constitutes a very robust FRET pair for many FRET techniques.

To avoid the interference with yeast cytosolic autofluorescence and to better visualize less abundant donor proteins, combinations of green and red fluorescent proteins (GFPs and RFPs) can be used as FRET donor–acceptor pairs. Though EGFP still has good fluorescent characteristics to serve as a bright FRET donor, mNG offers substantially higher brightness and better spectral overlap with RFP-based acceptors. Compared with many RFPs tested in yeast, mCherry and mScarlet-I [94] represent, in our opinion, the most suitable FRET acceptors because of their good and excellent brightness, respectively. However, though EGFP-mCherry and mNG-mScarlet-I FRET pairs are valuable for acceptor photobleaching, both mCherry and mScarlet-I come with some limitations for sensitized emission FRET techniques. First, the fluorescence of mCherry is too weak to be reliably monitored by ratiometric FRET. Second, though mScarlet-I is substantially brighter than mCherry and its ratiometric readout is, thus, possible, it is clearly less photostable. Careful correction for its photobleaching should be, therefore, applied for time-lapse sensitized emission experiments.

The power of yeast genetics and available tagging techniques offer multiple ways to fuse a suitable FP to a protein of interest. These techniques allow endogenous tagging of proteins, either N- or C-terminally (or even internally), in a fast and efficient way, often directly providing short linkers needed for the random orientation of the FP in the protein fusion. The full coverage of these techniques is beyond the scope of this review, so we list here only selected original works focused on FP tagging [95,96,97,98,99]. Similarly, the palette of tools for well-tuned expression of FRET biosensors from either a plasmid or from the genome is broad, so we suggest first testing an expression system used in the original study or established in your lab.

We conclude with several practical tips regarding cell and microscope preparation for FRET imaging in yeast. As autofluorescence of yeast cytosol and vacuoles can seriously interfere with the fluorescence signals of especially CFPs and RFPs, respectively, we suggest minimizing it by using a low-fluorescence minimal medium (without folic acid, riboflavin, and eventually tryptophan) and working with freshly grown early logarithmic cells. Also, movement of cells during imaging should be prevented by their stable attachment to the microscopy slide (e.g., by lectin concanavalin A). For extensive FRET experiments, it is also worth optimizing the optical configuration of the microscope (used excitation or emission filters and dichroic mirrors) to achieve the highest performance of your chosen FRET pair (e.g., minimized bleed-through and cross-talk signals). Finally, considering that in vivo FRET signal often constitutes only a small percentage of the total registered fluorescence, a careful control of its specificity is always desirable. This is imperative especially when the FRET donor and acceptor are two molecules of different abundance or mobility, as this can influence apparent FRET values (discussed in more detail in [79]). All necessary control strains (donor or acceptor only, no FRET control with clearly separated donor–acceptor pair, FRET positive donor–acceptor FPs fusions, etc.), including strains expressing protein fusions with interchanged FPs, should be considered to corroborate observed FRET signals.

## 6. Concluding Remarks

The outstanding ability to sensitively and non-invasively analyze molecular proximities and reactions directly in living cells has made FRET microscopy an invaluable tool for modern biomedical research. The importance and application of FRET in yeast will probably be ever-increasing, especially if it is coupled to current single molecule localization microscopy approaches [21], to which FRET is not only partially complementary, but even superior in regard to spatial resolution. In addition, FRET will certainly gain from current fast development of new microscopy tools and fluorescent probes. All of the above, applied on the experimentally robust and outstandingly characterized yeast models, gives strong promise that FRET studies will provide us with a great wealth of new mechanistic and systems information about fundamental cellular processes, often directly important for human wellness.

## Figures and Tables

**Table 1:** Protein complexes analyzed by FRET in yeast.

Protein Complex	FRET Technique ^1,2^	FRET Donor–Acceptor ^1,2^	References
Nuclear pore complex (NPC)	sensitized emission	CFP-YFP	[11,12,42]
Spindle pole body (SPB)	sensitized emission acceptor photobleaching	CFP-YFP mTq2-YFP	[16,17,18,19,20]
Kinetochore	sensitized emission FLIM	GFP-mCherry mTq2-YFP	[13,14,15]
Contractile ring	acceptor photobleaching	GFP/mNG-mCherry	[21]
Endocytic coat	acceptor photobleaching	GFP-mCherry mTq-mNG mNG-mScarlet	[22]
Cohesin	sensitized emission	CFP-YFP	[24]
SAGA-Gal4 transcription factor	acceptor photobleaching spectral FRET	CFP-YFP	[25,43]
PCNA-SAS-I complex	FLIM	CFP-YFP	[26]
ATR complex Dcp2-Mec1- PP4 phosphatase Psy2-Php3	sensitized emission	GFP-RFP	[27]
Ste2 oligomerization	spectral FRET	CFP/GFP-YFP	[28,29,30,44]
Fet3-Ftr1 iron permease	spectral FRET	CFP-YFP	[31]
Ctr1 transporter oligomerization and copper binding	spectral FRET	CFP-YFP	[32]
Ato1-Ato2 proteins	acceptor photobleaching, FLIM	GFP-tdimer2 CFP-Venus	[33]
V-ATPase disassembly	sensitized emission	CFP-YFP	[34]
Tom70 oligomerization	sensitized emission	CFP-YFP	[35]
CDK inhibitor Sic1-cyclins	FLIM	mCerulean-YFP	[36]
Ste5-Fus3 interaction	acceptor photobleaching	GFP-mStrawberry	[37]
Prp prion aggregation	donor photobleaching	CFP-YFP	[38]
Prp-amyloid β interaction	acceptor photobleaching	CFP-YFP	[39]
HTT huntingtin aggregation	acceptor photobleaching	CFP-Venus	[40]
Toh1 aggregation with Rnq1 and Sup35 prion proteins	acceptor photobleaching	CFP-YFP	[41]

^1^ For individual FRET techniques and fluorescent proteins, see please the main text. ^2^ CFP, cyan fluorescent protein; YFP, yellow fluorescent protein; mTq2, mTurquoise2; FLIM, fluorescence lifetime imaging microscopy; GFP, green fluorescent protein; mNG, mNeonGreen.

**Table 2:** FRET biosensors in yeast.

Studied Analyte/Process	Sensor Name (Sensor Origin)	FRET Donor–Acceptor (FRET Method) ^1^	References
Maltose	FLIPmal (MBP)	CFP-YFP (spectral FRET)	[67,68]
Glucose (Galactose)	FLIPglu sensors (MglD)	CFP-Venus	[69,70]
Trehalose-6P	T6P-TRACKs (TreR)	CFP-Venus	[71]
ATP	AT1.03 sensors (ε subunit of FoF1-ATP synthase)	CFP-Venus	[69,72,73]
Histidine	FLIP-cpHisJ194 (HisJ)	CFP-Venus	[74]
Lysine	FLIPK (LAO)	CFP-YFP	[75]
Zinc ion	ZF1/2, ZF3/4, ZapCY1/2 (Zap1)	CFP-YFP/Citrine	[52,53]
Redox state	Redoxfluor (Yap1)	Cerulean-Citrine	[50,55]
Oxygen	YFOS (FbFP)	FbFP-YFP (spectral FRET)	[76]
Nitrate Oligopeptides	NiTrac sensors, PepTrac sensors	mCerulean-Aphrodite (spectral FRET)	[56]
Abscisic acid	ABACUS1 sensors	Cerulean-Citrine	[57]
Prion proteins nucleation	AmFRET	mEos3.1 (FACS)	[58]
Amyloid β cleavage by evolved protease	PrECISE	CyPet-Ypet (FACS)	[59]
PolII promoter activity	IMAGEtags (RNA aptamers)	Cy3-Cy5 (sensitized emission)	[60]
MAPK signaling pathway	yEKAREV	CFP-YPet	[61]
cAMP/PKA signaling pathway	Epac2-camps (Epac2) AKAR3	CFP-YFP CFP-cpVenus	[62]
Force for chromosome segregation	Ndc80 tension sensor	CFP-YPet	[63]
Force for endocytic vesicle formation	molecular tension sensors in Sla2 protein	mTq2-mNG	[64,66]

^1^ If a method other than ratiometric FRET was used, it is specified in parentheses.

**Table 3:** Comparison of FRET microscopy techniques.

FRET Technique	Advantages	Disadvantages
acceptor photobleaching	easy to set up and calculate	endpoint assay (time-lapse measurements only indirectly)
sensitized emission (including ratiometric FRET)	easy to set up time-lapse measurements	acquisition of controls necessary for FRET calculation (not for ratiometric FRET)
FRET-FLIM	pools of FRET-involved/absent molecules discernable time-lapse measurements	complex setup and data analysis
